# Unveiling a Rare Side Effect: A Report of a Unique Case of Second-Degree Type 2 Sinoatrial Node Exit Block Induced by Adenosine Infusion

**DOI:** 10.7759/cureus.53310

**Published:** 2024-01-31

**Authors:** Dibyasundar Mahanta, Anup K Budhia, Rama Chandra Barik, Debasish Das, Debasis Acharya

**Affiliations:** 1 Cardiology, Institute of Medical Sciences (IMS) SUM Medical College and Hospital, Bhubaneswar, IND; 2 Internal Medicine, Hi-Tech Medical College and Hospital, Bhubaneswar, IND; 3 Cardiology, All India Institute of Medical Sciences, Bhubaneswar, Bhubaneswar, IND; 4 Cardiology, Hi-Tech Medical College and Hospital, Bhubaneswar, IND

**Keywords:** negative chronotropy, junctional bradycardia, his bundle, paroxysmal supraventricular tachycardia, atrio ventricular block

## Abstract

Adenosine is a widely used pharmacologic agent in the field of cardiology, predominantly for the termination of supraventricular tachycardias and diagnostic purposes. Most of the side effects are short-lasting due to its very short half-life. Fatal complications of adenosine are rare but can include ventricular fibrillation, ventricular tachycardia, and asystole. Proper medical supervision and monitoring are crucial to minimize risks. We report a unique case of a second-degree type 2 sinoatrial node exit block following intravenous adenosine administration in a 25-year-old male presenting with palpitations.

## Introduction

Adenosine is an endogenous purine nucleoside that forms when adenine triphosphate (ATP) breaks down. Adenosine is typically more stable than ATP; therefore, it is primarily used as a therapeutic drug and for diagnostic purposes by cardiologists, electrophysiologists, and emergency physicians [1]. The most common therapeutic use of adenosine is to terminate supraventricular tachycardia (SVT). Side effects of adenosine are short-lived, as its half-life is very short (10 seconds). A transient atrioventricular (AV) delay or block is a common manifestation, and this property of adenosine is responsible for termination and exploring the mechanism of tachycardia. However, when prolonged, it becomes a side effect that can rarely cause ventricular tachycardia, ventricular fibrillation, or cardiac arrest. Another common side effect is transient sinus bradycardia. A transient sinus pause may also occur less commonly due to the adenosine effect on the sinus node [2]. The sinus exit block has not been reported to date as a side effect. We report a case of adenosine-induced Mobitz type 2 second-degree sinus exit block in a 25-year-old patient who presented with recurrent episodes of palpitation.

## Case presentation

**Figure 1:**
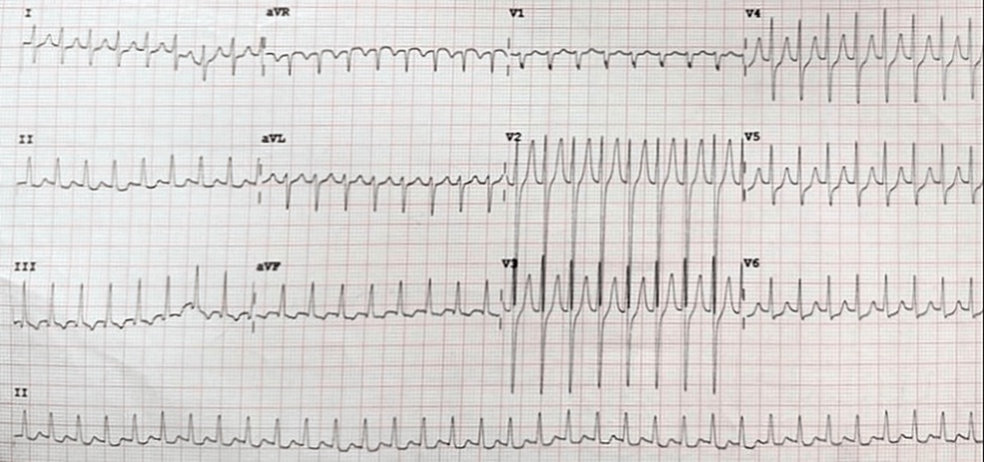
ECG during tachycardia ECG shows regular and narrow complex tachycardia at a rate of 190 beats per minute ECG, electrocardiogram

**Figure 2:**
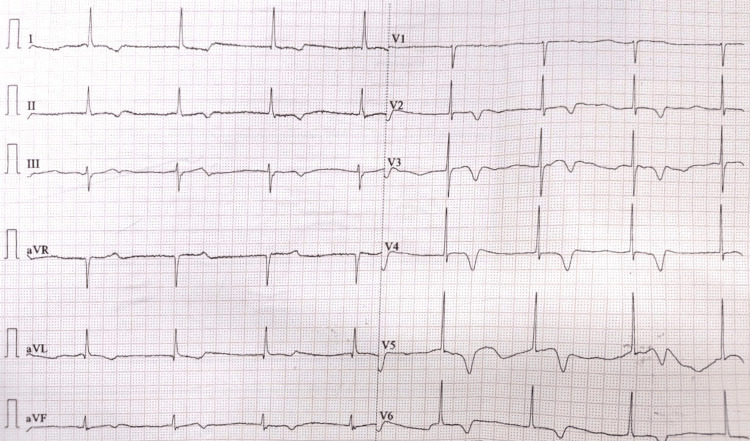
Immediate post-cardioversion ECG. Immediate post-cardioversion ECG shows junctional escape rhythm with a heart rate of 46 beats per minutes, and deep asymmetric T wave suggestive of memory T wave. ECG, electrocardiogram

**Figure 3:**
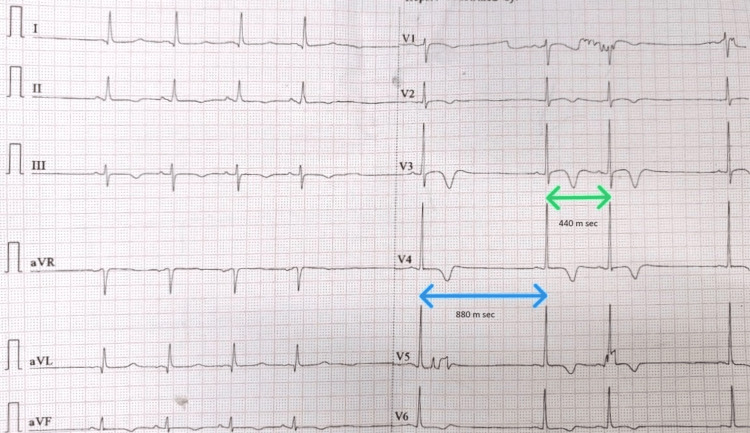
Second post-cardioversion ECG The second post-cardioversion ECG shows sinus rhythm, with intermittent pauses. The PP interval containing the pause (the blue arrow is 44 small squares or 1760 msec) is exactly twice the basic PP interval (the green arrow is 22 small squares or 880 msec), suggesting a second-degree Mobitz type 2 sinoatrial exit block. ECG, electrocardiogram

A 25-year-old male with a history of recurrent palpitations for the last six months presented to the emergency department with a similar complaint for one hour. An electrocardiogram (ECG) showed a narrow QRS complex and regular tachycardia (Figure 1), suggestive of SVT. After obtaining consent and keeping the defibrillator ready by the patient's side, 6 mg of adenosine was infused rapidly through a central vein. Tachycardia was terminated, and the immediate post-cardioversion ECG (Figure 2) showed junctional escape rhythm with a heart rate of 46 beats per minute and deep asymmetric T-wave inversion (memory T wave). A few minutes later, the ECG showed sinus rhythm with an intermittent pause (Figure 3). The PP interval intervening the pause was precisely twice that of the basic PP interval (the PP interval containing the pause is 44 small squares, or 1,760 msec, and the basic PP interval is 22 small squares, or 880 msec), suggesting a second-degree Mobitz type 2 sinoatrial (SA) node exit block. Subsequent ECG showed the disappearance of pause and memory T waves. The echocardiogram showed a structurally normal heart. All routine blood tests, including serum electrolytes, were within normal limits. The 72-hour Holter study revealed no evidence of sinus node dysfunction. An electrophysiology study (corrected sinus node recovery time) revealed normal sinus node function. Typical slow-fast atrioventricular node re-entrant tachycardia (AVNRT) was induced, and slow pathway modification was done.

## Discussion

Around one-third of patients receiving adenosine infusions develop clinical side effects that last for less than 60 seconds [3]. Facial flushing, dyspnea, and chest discomfort are the common side effects. They are generally mild and do not require intervention. Few cases of ventricular tachycardia and ventricular fibrillation have been reported, and these patients on evaluation were found to have acquired or congenital long QT syndrome and bypass tract [3,4]. Adenosine can shorten atrial action potentials, leading to a decrease in the effective refractory period. This process promotes the development of atrial flutter and fibrillation in a few patients [5]. Adenosine, by stimulating the carotid chemoreceptor, causes a reflex increase in sympathetic drive and serum catecholamine levels, and this results in transient sinus tachycardia and atrial or ventricular ectopy. These ectopics sometimes initiate re-entrant arrhythmia in patients with structural heart disease [5]. In addition, transient sinus bradycardia and arrest may also occur. Adenosine, through the A1 receptor, mainly affects the SA node, AV node, and atrium. It has little effect on the ventricle. Adenosine increases K (potassium) efflux out of SA nodal cells by directly inhibiting the I K_Ach_ receptor and indirectly inhibiting hyperpolarization-activated I_f_ (funny current) by activating cAMP (Cyclic adenosine monophosphate). This causes hyperpolarization of SA nodal cells, causing negative chronotropy [4]. Excess hyperpolarization causes severe sinus bradycardia and sinus pause. Adenosine causes hyperpolarization of the AV nodal cell by inhibiting I K_Ach_, causing negative dromotropy, which in excess leads to high-grade AV block. Prolonged sinus arrest or AV block occurs rarely, is dose-dependent, and is seen in patients with underlying conduction disturbances [5]. Adenosine sensitivity varies across cardiac pacemaker tissues, with the His bundle having the highest sensitivity and the SA node having the lowest sensitivity [6]. Therefore, sinus bradycardia and arrest are less common than AV block. Patients with sick sinus syndrome are more likely to develop prolonged pauses or asystoles [5]. Studies suggest that increased age may augment the sensitivity of adenosine receptors and impair adenosine transport [6]. Therefore, precaution is to be taken with older patients, and lower doses should be used if the drug is administered centrally.

## Conclusions

To our knowledge, a sinus exit block has not been reported to date. Again, our case is unique as our patient is young, received only 6 mg of adenosine, was not on drugs that potentiate the action of adenosine, and had a normal sinus node function in the electrophysiology study. However, the junctional rhythm and sinus exit block persisted for about five minutes. The junctional bradycardia may be due to prolonged sinus arrest or third-degree sinus exit block, and differentiation between the two conditions is difficult. As the patient later developed a second-degree sinus exit block, junctional bradycardia is probably due to a third-degree SA exit block. Future studies and case reports are crucial in enhancing our understanding of these rare side effects and optimizing patient safety during adenosine administration.
